# The relationship between dietary intake of ω-3 and ω-6 fatty acids and frailty risk in middle-aged and elderly individuals: a cross-sectional study from NHANES

**DOI:** 10.3389/fnut.2024.1377910

**Published:** 2024-05-09

**Authors:** Zhaoqi Yan, Yifeng Xu, Keke Li, Wenqiang Zhang, Liangji Liu

**Affiliations:** ^1^Graduate School, Jiangxi University of Traditional Chinese Medicine, Nanchang, China; ^2^Department of Respiratory and Critical Care Medicine, Jiangxi Hospital of Integrated Traditional Chinese and Western Medicine, Nanchang, China; ^3^Department of Respiratory and Critical Care Medicine, Affiliated Hospital of Jiangxi University of Traditional Chinese Medicine, Nanchang, China

**Keywords:** dietary ω-3 fatty acids, dietary ω-6 fatty acids, frailty, national health and nutrition examination survey, cross-sectional study

## Abstract

**Background:**

Frailty is a complex clinical syndrome characterized by a decline in the functioning of multiple body systems and reduced adaptability to external stressors. Dietary ω-3 fatty acids are considered beneficial dietary nutrients for preventing frailty due to their anti-inflammatory and immune-regulating properties. However, previous research has yielded conflicting results, and the association between ω-6 fatty acids, the ω-6: ω-3 ratio, and frailty remains unclear. This study aims to explore the relationship between these factors using the National Health and Nutrition Examination Survey (NHANES) database.

**Materials and methods:**

Specialized weighted complex survey design analysis software was employed to analyze data from the 2005–2014 NHANES, which included 12,315 participants. Multivariate logistic regression models and restricted cubic splines (RCS) were utilized to assess the relationship between omega intake and frailty risk in all participants. Additionally, a nomogram model for predicting frailty risk was developed based on risk factors. The reliability of the clinical model was determined by the area under the receiver operating characteristic (ROC) curve, calibration curves, and decision curve analysis (DCA).

**Results:**

In dietary ω-3 intake, compared to the T1 group (≤1.175 g/d), the T3 group’s intake level (>2.050 g/d) was associated with approximately 17% reduction in frailty risk in model 3, after rigorous covariate adjustments (odds ratio (OR) = 0.83, 95% confidence interval (CI): (0.70, 0.99)). In dietary ω-6 intake, the T2 group’s intake level (>11.423, ≤19.160 g/d) was associated with a 14% reduction in frailty risk compared to the T1 group (≤11.423 g/d) (OR: 0.86, 95% CI: 0.75, 1.00, *p* = 0.044). RCS results indicated a non-linear association between ω-3 and ω-6 intake and frailty risk. Both ROC and DCA curves demonstrated the stability of the constructed model and the effectiveness of an omega-rich diet in reducing frailty risk. However, we did not find a significant association between the ω-6: ω-3 ratio and frailty.

**Conclusion:**

This study provides support for the notion that a high intake of ω-3 and a moderate intake of ω-6 may contribute to reducing frailty risk in middle-aged and elderly individuals.

## Introduction

Frailty is a comprehensive syndrome characterized by a decline in physiological reserves when confronted with external stressors. Individuals in a frail state experience decreased capabilities in areas such as musculoskeletal function, nutritional intake, metabolism, cognition, and the nervous system. Frailty is also associated with a higher risk of adverse outcomes, including falls, fractures, hospitalization, and disability (1). Heterogeneity exists among individuals in terms of frailty, and it is not simply a linear function of age. Frailty shows a significant association with Disability-adjusted life years (DALYs) compared to age alone (2), making it advantageous for assessing an individual’s health status (3). Currently, due to societal stress and unhealthy lifestyles, many middle-aged individuals are also experiencing frailty, which is no longer exclusively considered a complex condition associated only with the elderly. It is now viewed as a manifestation of physical decline, thereby increasing the urgency for frailty prevention strategies (4). To date, there is no cure for frailty, making prevention and symptom management the desired goals. The most effective interventions involve increased physical activity and maintaining a balanced nutritional diet (5, 6).

Polyunsaturated fatty acids (PUFAs) are essential fats that must be obtained from external sources (7). Among these, ω-3 and ω-6 fatty acids (omega-3 and omega-6) are the two main families of PUFAs and play crucial roles in heart health, brain development and function, inflammation regulation, immune system support, mental health, and cancer prevention, among other aspects (8, 9). ω-3, in particular, is considered to have a direct impact on factors closely associated with frailty due to its anti-inflammatory properties and its role in preserving muscle and bone health (10, 11), making it a potential risk reducer for frailty (6, 12). Studies by León-Muñoz (13) and Hutchins-Wiese (14) have confirmed the benefits of eicosapentaenoic acid (EPA) and docosahexaenoic acid (DHA), the primary components of ω-3, in improving frailty phenotypic symptoms. Additionally, a meta-analysis is working to provide guidance in dietary supplementation recommendations of omega for frailty prevention in the elderly (15). However, some large-scale cohort studies have contradictory findings (16, 17), and the data regarding ω-6 interventions for frailty are limited, impeding strong recommendations for the use of long-chain polyunsaturated fatty acids in frailty prevention.

There is currently no research clearly defining the specific intake levels of ω-3 and ω-6 and their association with frailty risk. Therefore, the aim of this study is to explore the correlation between dietary ω-3 and ω-6, as well as their ratio, and the risk of frailty using data from the National Health and Nutrition Examination Survey (NHANES). The NHANES study is a multi-stage, stratified, and nationally representative investigation of the US population conducted by the National Center for Health Statistics of the Centers for Disease Control and Prevention. Its objective is to assess the nutritional and health status of Americans, gathering data on demographics, dietary habits, physical examinations, laboratory tests, and questionnaires. This article is presented in accordance with the STROBE reporting checklist.

## Materials and methods

### Study population in NHANES

**Figure 1:**
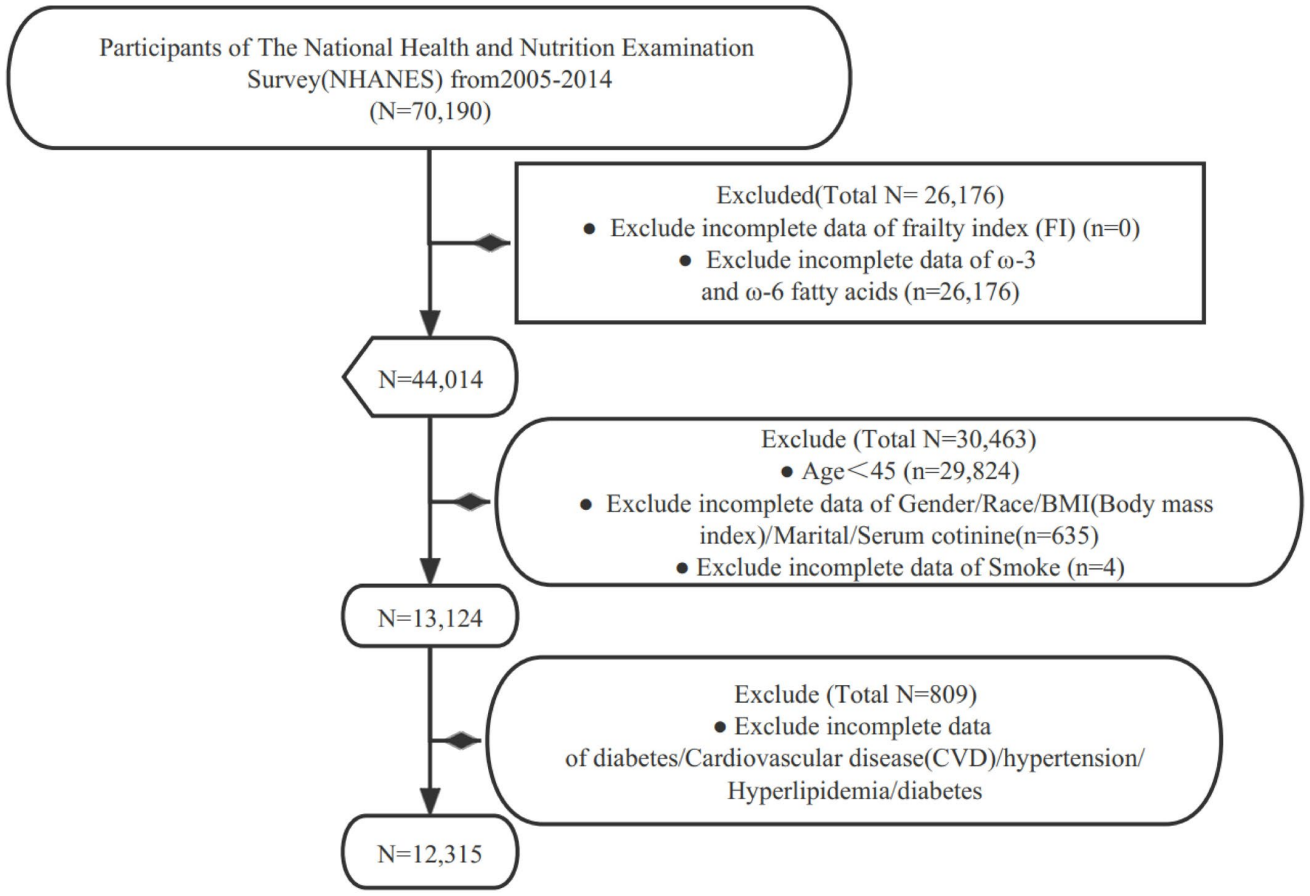
Inclusion flow diagram.

For our study, we specifically examined data collected between 2005 and 2014. Subjects were excluded from our study for the following reasons: (1) missing data on the frailty index (FI); (2) missing data on ω-3 and ω-6 intake; (3) aged under 45 years old (Non-Elderly Population); (4) missing covariate data (such as hypertension, hyperlipidemia, diabetes, etc.) (Figure 1).

### Frailty

Building upon the standards for frailty established by Searle (18), Hakeem (19) further expanded the FI to include 49 deficits, encompassing various systems. These systems include cognitive function (related to confusion and memory issues), dependence (difficulties with activities of daily living), depression (assessed with the Patient Health Questionnaire-9), comorbidities (various chronic diseases), hospital utilization, and self-rated health status (healthcare utilization frequency and prescription medication counts compared to the past year), physical performance and anthropometric measurements (grip strength and body mass index), and laboratory values (including complete blood counts and blood glucose levels). The FI is expressed as the number of deficits acquired by participants divided by the total potential deficits (with a numerical range from 0–1) (Supplementary Table S1). For example, an individual with 10 deficits would have an FI score of 0.20 (10/49 = 0.20). Consistent with previous research, we categorized individuals as “robust” (FI ≤ 0.21) and “frail” (FI > 0.21) (19).

### Dietary ω-3 and ω-6 intake

Dietary ω-3 and ω-6 intake was based on data obtained from 24-h dietary recall interviews. The primary dietary interview was conducted by trained interviewers at the Mobile Examination Center (MEC) using an automated data collection system. Detailed information on data processing procedures can be found in the NHANES website’s dietary interview component [The examination protocol and data collection methods. https://wwwn.cdc.gov/nchs/data/nhanes/2011-2012/manuals/mec_in_person_dietary_procedures_manual_jan_2012.pdf].

In this study, we aimed to capture as many components of ω-3 and ω-6 as possible. Dietary ω-3 included alpha-linolenic acid (ALA, 18:3), EPA (20:5), docosapentaenoic acid (DPA, 22:5), and docosahexaenoic acid (DHA, 22:6), in addition to other forms [stearidonic acid (SDA), 18:4]. Dietary ω-6 included linoleic acid (LA, 18:2) and arachidonic acid (AA, 20:4) (20). Daily average intake of ω-3 and ω-6 was calculated from dietary intake based on the USDA Food and Nutrient Database for Dietary Studies, and only dietary intake of these fatty acids was considered, with supplements not being taken into account [U.S. Department of Agriculture, Agricultural Research Service. Usda Food and Nutrient Database for Dietary Studies. https://www.ars.usda.gov/northeast-area/beltsville-md-bhnrc/beltsville-human-nutrition-research-center/food-surveys-research-group/docs/wweianhanes-overview/].

Furthermore, the low-fat dietary pattern is defined based on the total lipid intake and total calorie intake of the respondents. Specifically, this dietary pattern is characterized by a total lipid intake of less than or equal to 30% of the daily intake. Since the NHANES questionnaire does not provide a detailed breakdown of all lipid energies, we used a fat energy coefficient for conversion, where approximately 1 g of fat intake equals 9 kcal.

### Covariates

Covariates included age, sex, race/ethnicity (Mexican American, other Hispanic, non-Hispanic white, non-Hispanic black, non-Hispanic Asian, other race), BMI, marital status, smoking status, cotinine, hypertension, hyperlipidemia, diabetes and coronary artery disease (CVD). BMI was divided into three categories: Normal (<25 kg/m^2^), Overweight (≥25 kg/m^2^, <30 kg/m^2^), and Obese (≥30 kg/m^2^). Smoking status was classified as Current Smoker (Defined as having smoked more than 100 cigarettes in a lifetime and still smoking), Former Smoker (Defined as having smoked more than 100 cigarettes in a lifetime but no longer smoking), Never smoke (defined as smoking less than 100 cigarettes in their lifetime). Hypertension was defined according to the American Heart Association/American College of Cardiology (AHA/ACC) 2017 guidelines as systolic blood pressure ≥ 130 mmHg or diastolic blood pressure ≥ 80 mmHg and self-reported diagnosis or use of antihypertensive medication. As per the guidelines set by the Adult Treatment Panel III (ATP 3) of the National Cholesterol Education Program (NCEP), hyperlipidemia is defined by the following criteria: total cholesterol levels equal to or exceeding 200 mg/dL, triglyceride levels equal to or exceeding 150 mg/dL, HDL cholesterol levels below 40 mg/dL for men and below 50 mg/dL for women, or LDL cholesterol levels equal to or exceeding 130 mg/dL (21). Diabete as any of the following: (1) HbA1c levels equal to or greater than 6.5%; (2) serum glucose levels exceeding 200 mg/dL at 2 h after a 75 g glucose load (OGTT); (3) fasting glucose levels equal to or greater than 126 mg/dL; (4) self-reported diagnosis of diabetes; (5) self-reported use of insulin or other diabetes medication. The duration of diabetes was determined by subtracting the participant’s current age from the self-reported age at diagnosis, or zero for individuals diagnosed during the NHANES examination. For CVD, a positive response to any of the following questions was defined as CVD: “Has a doctor or other health professional ever told you that you have congestive heart failure (CHF)/coronary heart disease (CHD)/angina/heart attack/stroke?”

Considering drugs affecting lipid metabolism, we defined lipid-lowering drugs and drug categories (such as statins, fibric acid derivatives, ezetimibe, cholesterol absorption inhibitors, and PCSK9 inhibitors, etc.) using the Multum Lexicon standardized drug codes or therapeutic classification schemes. Specifically, we confirmed the use of prescription drugs in the past month, with primary treatment category drugs classified as “metabolism modifiers” (code “358”) and secondary treatment drug category classified as anti-hyperlipidemic agents (code “19”), and associated them with respondents’ individual identification symbols “SEQN” through the unique identifier “RXDDRGID” (https://wwwn.cdc.gov/Nchs/Nhanes/1999-2000/RXQ_DRUG.htm#Component_Description).

### NHANES analysis

A complex sampling design was implemented to ensure nationally representative estimates. All analyses were adjusted for survey design and weighted variables, with new sample weights calculated as the original 2-year sample weights divided by 2. Dietary ω-3 and ω-6 fatty acids intake were categorized into tertiles (with 1/3, 2/3, and 1 as cut-off points). The ω-6: ω-3 ratio was divided into four groups: recommended (≤4), mildly high (>4, ≤10), high (>10, ≤15), and very high (>15). Continuous variables were expressed as mean ± standard deviation (SD), while categorical variables were presented as counts (N) and percentages (%). Weighted t-tests (for continuous variables) and weighted chi-square tests (for categorical variables) were used to assess differences between robust and frailty subjects. Kruskal-Wallis tests (for continuous variables) or weighted chi-square tests (for categorical variables) were employed to evaluate differences among the three groups based on omega intake.

Initially, a crude model was fitted, followed by stepwise adjustment for covariates. Model 1 adjusted for age, sex, and race; Model 2 further adjusted for BMI, marital status, serum cotinine, and smoking status based on Model 1; Model 3 additionally adjusted for hyperlipidemia, hypertension, diabetes, and CVD based on Model 2. Results were presented as odds ratios (OR) with corresponding 95% confidence intervals (95% CI). Subgroup analyses were conducted for significant results. Furthermore, a logistic regression model was used to assess the significance of the interaction between omega intake and covariates on frailty. Regression models and restricted cubic splines (RCS) flexibly model the relationship between independent and dependent variables, especially in regression analysis. When the relationship between independent and dependent variables is not a simple linear one, RCS can help capture this non-linear relationship. They allow researchers to approximate the relationship using different polynomial functions within different ranges of independent variables, thereby providing a more accurate description of the data’s trend. RCS with three knots, at the 10^th^, 50^th^, and 90^th^ percentages, were used to explore the non-linear relationships of Omega intake levels and frailty in the linear terms model.

In addition, the risk magnitude of all variables on frailty was evaluated by constructing a nomogram. Subsequently, calibration curves were plotted to assess the fit between the predicted probabilities from the nomogram and the actual proportions. To further evaluate the sensitivity of the constructed model, the performance of the model was assessed using the area under the curve (AUC) of the receiver operating characteristic (ROC) curve. Decision curve analysis (DCA) was employed to estimate the net benefit at different threshold probabilities, determining the clinical utility of the model (22). Statistical significance was considered at a *p*-value <0.05, and all reported probability tests were two-sided.

## Results

### The baseline characteristics of the participants

**Figure 2:**
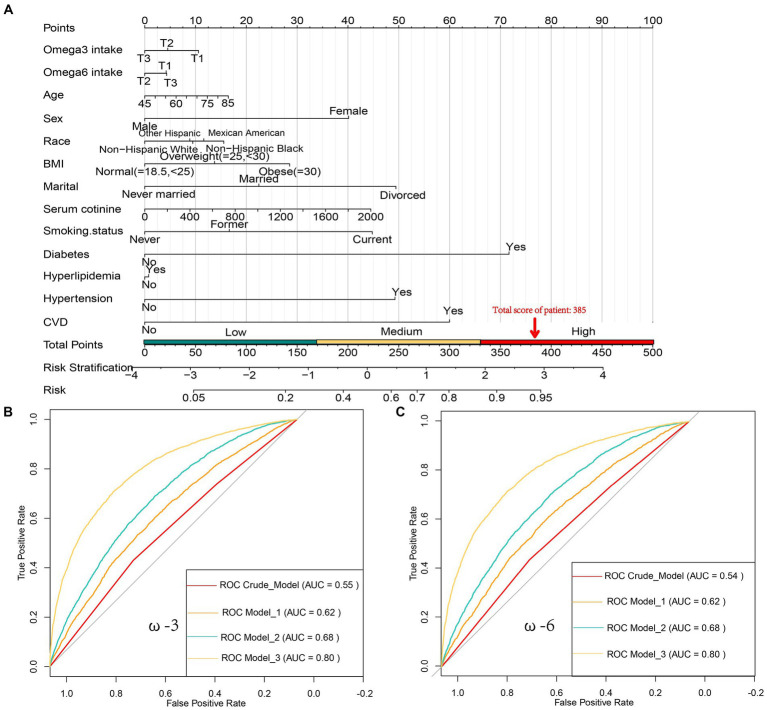
**(A)** Nomogram for Predicting Frailty Risk, used to assess the risk of frailty based on factors such as Age, Sex, Race, Marital Status, BMI, serum cotinine levels, Smoking status, Hypertension, Hyperlipidemia, Diabetes, daily intake of ω-3 [T1 (≤1.175 g/d), T2 (1.175<, ≤2.050 g/d), T3 (>2.050 g/d)], and daily intake of ω-6 [T1 (≤11.423 g/d), T2 (11.423<, ≤19.160 g/d), T3 (>19.160 g/d)]. Each predictor has a score, and the total score represents the likelihood of frailty. For example, an 85-year-old male, Non-Hispanic Black, obese, divorced, with a serum cotinine level of 1800, currently smoking, and having diabetes but not high cholesterol, high blood pressure, or coronary artery disease (CVD), with daily intake of 1 g ω-3 and 15 g ω-6, would have a frailty score of 385 (16 + 40 + 15 + 28 + 49 + 40 + 45 + 0 + 0 + 72 + 10 + 0 = 315), indicating a frailty risk of over 90%; **(B)** Receiver Operating Characteristic (ROC) curves showing the area under the curve (AUC) for models related to daily ω-3 intake, including the Crude model, Model 1, Model 2, and Model 3, with AUC values of 0.62, 0.55, 0.68, and 0.80, respectively; **(C)** ROC curves showing the AUC for models related to daily ω-6 intake, including the Crude model, Model 1, Model 2, and Model 3, with AUC values of 0.62, 0.54, 0.68, and 0.80, respectively.

In this study, a total of 12,315 individuals were finally included. Based on the exclusion criteria, 3,568 participants were classified as “Frail.” Compared to the “Robust” group, which consisted of 8,747 individuals, the “Frail” individuals were older, had a higher proportion of females, obesity, divorced individuals, and smokers (smoking history population). Additionally, they had a higher prevalence of underlying conditions such as hypertension and hyperlipidemia. Their intake of ω-3 was notably inadequate, and their intake of ω-6 was slightly lower. However, the ω-6:ω-3 ratio was significantly high at 10.1, indicating a deficiency of ω-3 intake and an imbalance in the ω-6 to ω-3 ratio compared to the recommended values in the human evolutionary diet (Table 1). These findings align with the current health challenges faced by frail individuals, especially in terms of their dietary habits. These risk factors are visualized through a nomogram (Figure 2A). The daily intake of ω-3 was divided into three equal parts using the tertiles method: T1 (≤1.175 g/day), T2 (>1.175, ≤2.050 g/day), and T3 (>2.050 g/day). Similarly, the daily intake of ω-6 was divided into three parts: T1 (≤11.423 g/day), T2 (>11.423, ≤19.160 g/day), and T3 (>19.160 g/day). It was observed that female individuals, divorced individuals, smokers, and those with hypertension were less attentive to ω-3 and ω-6 intake. In contrast, individuals with normal BMI did not pay much attention to the intake of polyunsaturated fatty acids. Additionally, we also investigated the use of ω-3 dietary supplements, with approximately 5.3% of respondents reporting their intake. There was no statistically significant difference in the intake of supplements between the frail and robust groups, indicating that supplements are unlikely to have influenced the results of this study. It’s interesting to note that an increased intake of ω-3 fatty acids often goes hand in hand with an increased intake of ω-6 fatty acids, and the proportion of vulnerable populations is also on the decline. When grouping individuals based on the ω-6: ω-3 ratio, there was no significant difference in the proportion of frail individuals in these groups, and most variables showed no significant differences (Supplementary Tables S2–S5).

**Table 1:** Characteristics of participants by Robust or Frail. (NHANES 2005–2014, *N* = 12,315).

**Characteristic**	**Overall**, *N* = 12,315 (100%)^1^	**Robust**, *N* = 8,747 (77%)^1^	**Frail**, *N* = 3,568 (23%)^2^	***P* Value**
**Age (years)**	59.9 (10.6)	58.9 (10.2)	62.9 (11.5)	**<0.001**
**Sex**				**<0.001**
*Female*	6,305 (53%)	4,250 (51%)	2,055 (61%)	
*Male*	6,010 (47%)	4,497 (49%)	1,513 (39%)	
**Race**				**<0.001**
*Non-Hispanic White*	6,155 (76%)	4,445 (78%)	1,710 (71%)	
*Non-Hispanic Black*	2,606 (9.7%)	1,698 (8.3%)	908 (14%)	
*Mexican American*	1,678 (5.2%)	1,203 (5.0%)	475 (5.8%)	
*Other Hispanic*	1,064 (3.7%)	770 (3.6%)	294 (4.0%)	
*Other Race - Including Multi-Racial*	812 (5.1%)	631 (8.1%)	181 (5.2%)	
**BMI**				**<0.001**
*Normal* (≥18.5,<25)	3,067 (26%)	2,390 (29%)	677 (19%)	
*Obese* (≥30)	4,837 (38%)	3,045 (34%)	1,792 (51%)	
*Overweight* (≥25,<30)	4,411 (36%)	3,312 (37%)	1,099 (31%)	
**Marital**				**<0.001**
*Divorced*	7,129 (64%)	5,446 (68%)	1,683 (52%)	
*Married*	4,764 (34%)	3,049 (30%)	1,715 (44%)	
*Never married*	422 (2.2%)	252 (1.8%)	170 (3.6%)	
**Serum cotinine**	53 (127)	49 (123)	67 (136)	**<0.001**
**Smoking status**				**<0.001**
*Current*	2,123 (17%)	1,352 (15%)	771 (22%)	
*Former*	4,037 (32%)	2,777 (31%)	1,260 (35%)	
*Never*	6,155 (51%)	4,618 (54%)	1,537 (42%)	
**Hypertension**				**<0.001**
*Yes*	7,274 (53%)	4,476 (46%)	2,798 (76%)	
*No*	5,041 (47%)	4,271 (54%)	770 (24%)	
**Hyperlipidemia**				**<0.001**
*Yes*	10,074 (82%)	7,005 (80%)	3,069 (87%)	
*No*	2,241 (18%)	1,742 (20%)	499 (13%)	
**Diabetes**				**<0.001**
*Yes*	3,311 (20%)	1,605 (13%)	1,706 (43%)	
*No*	9,004 (80%)	7,142 (87%)	1,862 (57%)	
**CVD**				**<0.001**
*Yes*	10,203 (86%)	8,027 (93%)	2,176 (64%)	
*No*	2,112 (14%)	720 (7.1%)	1,392 (36%)	
**ω-3**	1.90 (1.37)	1.95 (1.39)	1.73 (1.30)	**<0.001**
*ALA*	1.65 (1.25)	1.69 (1.27)	1.53 (1.19)	**<0.001**
*SDA*	0.01 (0.04)	0.01 (0.04)	0.01 (0.03)	**<0.001**
*EPA*	0.09 (0.13)	0.09 (0.13)	0.06 (0.10)	**<0.001**
*DPA*	0.08 (0.04)	0.09 (0.04)	0.06 (0.04)	**<0.001**
*DHA*	0.07 (0.20)	0.08 (0.20)	0.05 (0.17)	**<0.001**
*T1* (≤1.175 g/d)	4,487 (33%)	2,953 (31%)	1,534 (40%)	
*T2* (>1.175, ≤2.050 g/d)	4,054 (33%)	2,952 (34%)	1,102 (32%)	
*T3* (>2.050 g/d)	3,774 (33%)	2,842 (35%)	932 (28%)	
**ω-6**	17 (11)	17 (11)	16 (11)	**<0.001**
*LA*	16 (11)	16 (11)	15 (11)	**<0.001**
*AA*	0.84 (0.13)	0.94 (0.13)	0.73 (0.13)	**0.001**
*T1* (≤11.423 g/d)	4,663 (33%)	3,106 (31%)	1,557 (40%)	
*T2* (>11.423, ≤19.160 g/d)	3,956 (33%)	2,882 (34%)	1,074 (31%)	
*T3* (>19.160 g/d)	3,696 (33%)	2,759 (35%)	937 (29%)	
**ω-6/ω-3**	10.1 (5.1)	10.1 (5.2)	10.1 (4.8)	0.8
*Recommended* (≤4)	264 (1.9%)	202 (2.0%)	62 (1.6%)	
*Mildly high* (>4, ≤10)	7,474 (60%)	5,316 (60%)	2,158 (61%)	
*High* (>10, ≤15)	3,534 (30%)	2,500 (30%)	1,034 (29%)	
*Very high* (>15)	1,043 (8.5%)	729 (8.5%)	314 (8.3%)	
**Energy (kcal/d)**	2,035 (892)	2,083 (890)	1,877 (879)	**<0.001**
**Fat intake (g/d)**	79 (44)	81 (44)	73 (44)	**<0.001**

^1^Mean ± SD for continuous; n (%) for categorical; ^2^chi-squared test with Rao & Scott’s second-order correction. ω-3: omega-3 fatty acids; ω-6: omega-6 fatty acids.

### Associations between omega intake and frailty outcomes

Through the construction of multiple linear regression models, we found that for ω-3, using the daily intake level T1 (≤1.175 g/day) as the reference, at the T3 level of ω-3 intake (>2.050 g/day), there was a significant negative correlation with the risk of frailty in all models (*p* < 0.05). This result remained robust even after adjusting for covariates, especially after adjusting for variables such as age, gender, race, marital status, serum cotinine, smoking, hypertension, hyperlipidemia, CVD, and diabetes (Model 3). The risk reduction was up to 17% (OR: 0.83, 95% CI: 0.70–0.99, *p* = 0.035). Similarly, for ω-6, using the daily intake level T1 (≤11.423 g/day) as the reference, at the T2 level of ω-6 intake (>11.423, ≤19.160 g/day), there was a significant negative correlation with the risk of frailty in all models. In Model 3, the risk reduction was up to 14% (OR: 0.86, 95% CI: 0.75–1.00, *p* = 0.044). These results showed little variation across different models (Table 2).

**Table 2:** Weighted multivariate adjusted logistic regression analysis of frailty risk with different omega intake levels in NHANES from 2005 to 2014.

**Regression model**	**Crude model** **OR (95% CI)**	**Model 1** **OR (95% CI)**	**Model 2** **OR (95% CI)**	**Model3** **OR (95% CI)**
**ω-3 (g/day)**				
T1 (≤1.175)	Reference	Reference	Reference	Reference
T2 (>1.175, ≤2.050)	0.74 (0.65, 0.85) ***	0.80 (0.70, 0.92)**	0.83 (0.72, 0.95) **	0.87 (0.75, 1.02)
T3 (>2.050)	0.64 (0.55, 0.74)***	0.75 (0.64, 0.88)***	0.78 (0.66, 0.92)**	0.83 (0.70, 0.99)*
**ω-6 (g /day)**				
T1 (≤11.423)	Reference	Reference	Reference	Reference
T2 (>11.423, ≤19.160)	0.72 (0.64, 0.80) ***	0.81 (0.72, 0.91)***	0.83 (0.73, 0.94) **	0.86 (0.75, 1.00)*
T3 (>19.160)	0.65 (0.58, 0.73)***	0.81 (0.71, 0.92)**	0.82 (0.72, 0.93)**	0.87 (0.76, 1.00)
**ω-6: ω-3 ratio**				
Recommended (≤4)	Reference	Reference	Reference	Reference
Mildly high (>4, ≤10)	1.27 (0.83, 1.94)	1.19 (0.77, 1.83)	1.20 (0.76, 1.91)	1.37 (0.89, 2.09)
High (>10, ≤15)	1.35 (0.86, 2.11)	1.37 (0.85, 2.18)	1.31 (0.84, 2.04)	1.26 (0.79, 2.01)
Very high (>15)	1.29 (0.78, 2.12)	1.15 (0.73, 1.82)	1.11 (0.69, 1.80)	1.05 (0.62, 1.78)

*P < 0.05; **p < 0.01; ***p < 0.001.

Multiple logistic regression model: Model 1: adjusted for age, sex, race; Model 2: adjusted for age, sex, race, marital, serum cotinine, BMI, smoking status; Model 3: adjusted for age, sex, race, marital, serum cotinine, BMI, smoking status, hypertension, hyperlipidemia, diabetes, CVD. ω-3: omega-3 fatty acids; ω-6: omega-6 fatty acids.

**Figure 3:**
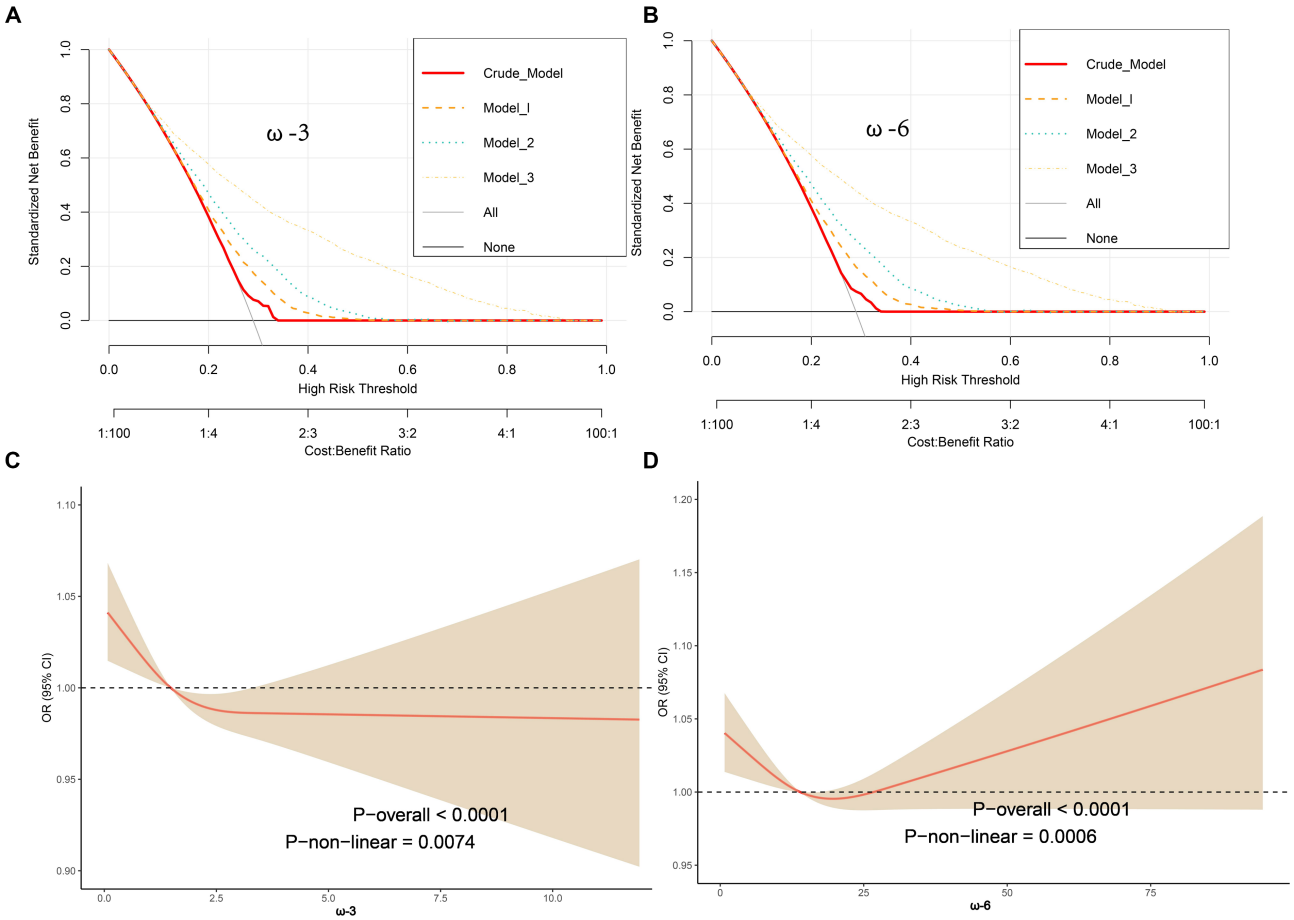
Decision Curve Analysis (DCA) showing net benefit curves for various models. The x-axis represents the threshold probability for frailty, and the y-axis represents net benefit. The red line, orange line, light blue line, and yellow line represent improved prediction nomograms for daily ω-3 and ω-6 intake, including the Crude model, Model 1, Model 2, and Model 3. The gray line represents the assumption that all patients use the nomogram model. The black line represents the assumption that no patients use the nomogram model to predict frailty risk. Based on the results from Table 2 and the DCA curves, it can be concluded that all constructed models can provide a net benefit for reducing frailty risk by increasing ω-3 intake (>2.050 g/d) **(A)** and by appropriately reducing ω-6 intake (>11.423, ≤19.160 g/d) **(B)**. The net benefit threshold is wide, and there are no side effects; adjustments were made using Restricted Cubic Spline (RCS) models for factors such as age, gender, race, BMI, marital status, serum cotinine, smoking, hypertension, hyperlipidemia, coronary artery disease, and diabetes to analyze the relationship between ω-3 and ω-6 and frailty. The solid red line represents the combined restricted cubic spline curve model, and the shaded area represents the 95% confidence interval for the combined curve. The dashed line represents the risk of frailty for different levels of ω-3 **(C)** and ω-6 **(D)** intake.

The ROC curves for ω-3 and ω-6 in Model 3 both had an AUC of 0.80, indicating that the constructed Model 3 had good predictive capability (Figures 2B,C). However, no significant association was found between the ω-6: ω-3 ratio and the reduction in frailty risk. Even when using a reference ratio of 4, the OR values in all models remained greater than 1, suggesting that increasing the ratio between the two is beneficial for reducing frailty development, though not reaching statistical significance. Further interaction analysis was performed on the covariates that showed significant differences between the healthy and frail groups in Table 1. Although there were differences between some subgroups, particularly in males, individuals with high BMI, non-smokers, low-fat intake diet, those without hypertension, diabetes, and those with cardiovascular diseases, these covariates showed no interaction with omega intake regarding frailty risk (*p* > 0.05). This suggests that the influence of covariates on the results of this study was minimal (Table 3). The decision curve analysis (DCA) showed that the net benefit probability for both ω-3 and ω-6 models in Model 3 ranged from 0 to 90%. The net benefit probability consistently remained higher than the group that received no intervention (depicted by the black solid line “None” in the graphs). This indicates that implementing the conditions in Model 3 (higher ω-3 intake and moderate ω-6 intake) is beneficial in reducing frailty risk without causing any side effects (Figures 3A,B). Furthermore, we used the RCS model to fit the relationship between ω-3 and ω-6 and frailty. After adjusting for covariates, a non-linear relationship was observed (*p* = 0.0085 for ω-3, *p* = 0.0006 for ω-6) (Figures 3C,D). To further assess the impact of drugs affecting lipid metabolism (primarily some lipid-lowering drugs) on this study, we conducted a sensitivity analysis by further grouping the population using these drugs. The results showed that in this study, there were 4,437 respondents with records of using these drugs in the past month. They still maintained a lower intake of ω-3 in the frail population but had a higher intake of ω-6 (Supplementary Table S6). After adjusting for multiple models, we still maintained the conclusion that a high level of ω-3 and moderate intake of ω-6 are associated with a reduced risk of frailty in middle-aged and elderly individuals (Supplementary Table S7).

**Table 3:** Subgroup analysis of the risk of frailty occurrence associated with Omega intake considering various confounding factors.

**Subgroup**	**ω-3** **Interaction *P*-Value**	**ω-3** **T2 (>1.175, ≤2.050)** **OR (95%CI)**	**ω-3** **T3 (>2.050)** **OR (95%CI)**	**ω-6** **Interaction *P*-Value**	**ω-6** **T2 (>11.423, ≤19.160)** **OR (95%CI)**	**ω-6** **T3 (>19.160)** **OR (95%CI)**
**Age**	*p* = 0.92			*p* = 0.65		
*45–60*		0.95 (0.74, 1.21)	0.84 (0.63, 1.11)		0.96 (0.75, 1.21)	0.90 (0.71, 1.14)
*61–75*		0.82 (0.67, 1.01)	0.79 (0.61, 1.02)		0.77 (0.61, 0.96)*	0.82 (0.64, 1.04)
*>75*		0.85 (0.64, 1.13)	0.87 (0.68, 1.11)		0.82 (0.62, 1.08)	0.87 (0.68, 1.12)
**Sex**	*p* = 0.54			*p* = 0.16		
*Female*		0.88 (0.74, 1.03)	0.90 (0.72, 1.12)		0.86 (0.71, 1.04)	0.98 (0.82, 1.19)
*Male*		0.86 (0.66, 1.12)	0.77 (0.61, 0.97)*		0.84 (0.66, 1.08)	0.74 (0.60, 0.93)**
**Race**	*p* = 0.62			*p* = 0.46		
*Non-Hispanic White*		0.78 (0.56, 1.08)	0.93 (0.68, 1.26)		0.89 (0.67, 1.17)	0.9 (0.65, 1.25)
*Non-Hispanic Black*		0.79 (0.63, 0.99)*	0.75 (0.60, 0.95)*		0.99 (0.79, 1.23)	0.97 (0.77, 1.23)
*Mexican American*		0.90 (0.74, 1.09)	0.83 (0.66, 1.05)		0.83 (0.69, 1.00)*	0.86 (0.72, 1.03)
*Other Hispanic*		0.99 (0.63, 1.57)	0.68 (0.41, 1.13)		0.76 (0.50, 1.16)	0.63 (0.37, 1.07)
*Other Race - Including Multi-Racial*		0.77 (0.43, 1.39)	1.11 (0.60, 2.06)		1.19 (0.65, 2.16)	0.90 (0.47, 1.73)
**Marital**	*p* = 0.53			*P* = 0.65		
*Divorced*		0.86 (0.70, 1.05)	0.89 (0.71, 1.12)		0.90 (0.74, 1.09)	0.90 (0.76, 1.07)
*Married*		0.90 (0.73, 1.10)	0.74 (0.57, 0.95)*		0.81 (0.65, 1.01)	0.80 (0.62, 1.03)
*Never married*		1.02 (0.48, 2.19)	1.13 (0.59, 2.19)		0.93 (0.42, 2.07)	1.59 (0.83, 3.03)
**BMI (Kg/m^2^)**	*p* = 0.72			*p* = 0.21		
*Normal* (<25)		0.91 (0.73, 1.15)	0.83 (0.62, 1.10)		1.05 (0.83, 1.31)	0.92 (0.73, 1.16)
*Obese* (≥30)		0.83 (0.66, 1.04)	0.92 (0.69, 1.22)		0.73 (0.57, 0.92)*	0.85 (0.63, 1.15)
*Overweight* (≥25,<30)		0.82 (0.56, 1.20)	0.62 (0.45, 0.86)**		0.72 (0.55, 0.94)*	0.68 (0.46, 0.98)*
**Smoking status**	*p* = 0.051			*p* = 0.35		
*Current*		1.15 (0.79, 1.68)	0.8 (0.60, 1.08)		0.89 (0.67, 1.18)	0.83 (0.63, 1.09)
*Former*		0.94 (0.74, 1.19)	0.95 (0.76, 1.19)		0.98 (0.76, 1.27)	0.91 (0.70, 1.18)
*Never*		0.74 (0.62, 0.89)**	0.8 (0.63, 1.01)		0.78 (0.64, 0.96)*	0.92 (0.76, 1.11)
**Hypertension**	*p* = 0.07			*p* = 0.51		
*Yes*		0.9 (0.76, 1.06)	0.94 (0.76, 1.17)		0.88 (0.75, 1.03)	0.92 (0.76, 1.12)
*No*		0.82 (0.61, 1.10)	0.63 (0.47, 0.85)**		0.81 (0.62, 1.06)	0.76 (0.58, 1.00)
**Hyperlipidemia**	*P* = 0.53			*P* = 0.53		
*Yes*		0.9 (0.77, 1.05)	0.84 (0.71, 1.01)		0.88 (0.74, 1.03)	0.90 (0.77, 1.04)
*No*		0.73 (0.51, 1.05)	0.76 (0.52, 1.12)		0.82 (0.56, 1.20)	0.74 (0.49, 1.11)
**CVD**	*P* = 0.51			*p* = 0.67		
*Yes*		0.84 (0.70, 1.02)	0.79 (0.64, 0.96)*		0.86 (0.72, 1.02)	0.84 (0.72, 0.99)*
*No*		0.98 (0.74, 1.30)	1.01 (0.74, 1.36)		0.87 (0.62, 1.22)	0.99 (0.72, 1.36)
**Diabetes**	*p* = 0.32			*p* = 0.37		
*Yes*		0.96 (0.73, 1.25)	0.93 (0.72, 1.22)		0.93 (0.71, 1.21)	0.94 (0.74, 1.19)
*No*		0.84 (0.71, 1.01)	0.78 (0.63, 0.96)*		0.83 (0.72, 0.97)*	0.83 (0.70, 0.99)*
**Fat intake level**	*p* = 0.10			*p* = 0.78		
*Low*		0.87 (0.69, 1.10)	0.57 (0.38, 0.85)**		0.95 (0.75, 1.21)	0.82 (0.52, 1.29)
*Non-low group*		0.90 (0.73, 1.11)	0.88 (0.71, 1.08)		0.84 (0.68, 1.04)	0.75 (0.62, 1.06)

Subgroup analysis adjustment factors: age; sex; race; education; marital; PIR; BMI; serum cotinine; smoking status; hyperlipidemia; hypertension; diabetes; CVD, excluding sub-group variables, and the reference object in the sub-group is ω-3-(omega-3 fatty acids)-T1 (≤1.175) and ω-6 (omega-6 fatty acids)-T1 (≤11.423). *P < 0.05; **p < 0.01.

## Discussion

As far as we know, this is the first large-scale study examining the levels of ω-3 and ω-6 intake and their ratio in relation to frailty. The findings of this study align with our expectations, indicating that high levels of ω-3 (>2.05 g/d) and moderate ω-6 intake (>11.423, ≤19.160 g/d) are associated with a reduced risk of frailty in middle-aged and older individuals. Furthermore, we observed for the first time that this association exhibits a nonlinear relationship.

With advancing age, the challenge of ensuring adequate nutrition for middle-aged and older individuals becomes more pronounced due to factors such as decreased appetite (age-related anorexia), physiological changes in the gastrointestinal system, oral health issues, swallowing difficulties, and medication use. Oxidative stress and inflammation are recognized as significant factors in the aging process (23). PUFAs (polyunsaturated fatty acids) can modulate antioxidant signaling pathways and regulate inflammatory processes (24), which potentially makes ω-3 intake beneficial for reducing frailty. Many pro-inflammatory cytokines are produced by fat cells and resident macrophages in adipose tissue, contributing to the pro-inflammatory state that underlies age-related diseases and frailty. Age-related muscle loss, which is a common part of the aging process, is linked to chronic low-grade inflammation, and a decrease in muscle mass is a key phenotype of frailty (25). Normal muscle mass starts to decline after the age of 40, with muscle function declining rapidly, up to 3% per year after the age of 60 (26). This reduction in muscle mass can lead to inconveniences in daily life for middle-aged and older individuals and contributes to additional risks such as falls, fractures, and heart failure (27, 28). In the context of the growing trend of Westernized diets, ω-3 appears to play a critical role in regulating inflammation and immune modulation (29, 30), as well as in maintaining muscle mass and function (31), all of which are important considerations in the context of frailty in middle-aged and older individuals. Research by León-Muñoz (13) suggests that daily supplementation of 2.4 grams of EPA and DHA, the primary components of ω-3, can improve physical functioning in frail individuals. Similarly, studies have shown that supplementing 1,500 mg/day of DHA and 1860 mg/day of EPA in healthy older men and women significantly increased thigh muscle volume by 3.6% and grip strength by 2.3 kg (32), while supplementing 720 mg/day of EPA and 40 mg/day of DHA had a positive impact on walking speed in older individuals (14). León-Muñoz also suggests that the addition of dietary antioxidants may synergize with ω-3 to improve physical functioning, an area that warrants further exploration. It’s important to note that some studies have reported different findings, such as the study by Orkaby (16), which found that taking 1 g/day of ω-3 did not affect frailty levels. However, this study only analyzed a single dose and did not include a control group with varying doses, while in our study, the ω-3 intake in the lowest range (T1) was less than 1.175 g/day, covering the range of Orkaby’s research. Similarly, studies by Krzymińska (33) and Rolland (34) examined the impact of lower doses of DHA and EPA on muscle strength and grip strength, which may explain the difference in results. This further supports the urgency of increasing daily ω-3 intake above 2.05 g.

Dietary ω-3, by itself, may potentially regulate muscle protein synthesis to maintain overall physical fitness (11). Additionally, since pro-inflammatory cytokines are associated with muscle atrophy (35), the anti-inflammatory effects of ω-3 make it a beneficial factor in preventing frailty. Another possibility is the close relationship between inflammation and apoptosis, the latter of which may be a biological pathway leading to muscle loss. For instance, tumor necrosis factor-alpha (TNF-α) induces muscle cell apoptosis (36), while ω-3 can inhibit TNF-α, interleukin-6 (IL-6), and C-reactive protein synthesis (CRP) (37, 38). LA in ω-6 plays a specific and unique role in maintaining the structural integrity and barrier function of human skin (39). The skin is the body’s natural first line of defense against various non-specific pathogens. Using LA as a partial substitute for saturated fatty acids is advantageous in reducing total cholesterol and low density lipoprotein cholesterol concentrations in the blood, which is likely to lower the risk of various underlying diseases (40, 41). AA, found in ω-6, is involved in mediating and regulating inflammatory responses (42), which are fundamental to inflammation. However, early-stage inflammatory responses are also related to the production of eicosatrienoic acid and subsequently to the induction of inflammation. Daily intake of 600 mg of AA can improve physical fitness (43), and Roberts (44) suggests that a daily intake of 1,000 mg of AA actually reduces inflammation. These complexities make our understanding of the role of ω-6 more nuanced. Moderate intake of ω-6 and an increase in ω-3 intake seem to be central axes in modulating the immune system and inflammatory responses for preventing frailty risk. This underscores the importance of maintaining nutritional balance and immune function in our bodies.

However, it’s worth noting that daily intake of over 2 g of ω-3 is challenging, and in most regions globally, this level of intake is not being achieved. Western diets tend to have an excess of ω-6 polyunsaturated fatty acids, often surpassing the recommended target range in this study (>11.423, ≤19.160 g/d) (8, 45), while ω-3 intake is often very low. This exposes individuals to a higher risk of frailty (46). Additionally, the high ω-3 levels in this study are also associated with high ω-6 intake, with only a small percentage of participants having a 1:1 ratio. This lack of a 1:1 ratio may have contributed to the inability to establish a direct association between ω-6/ω-3 levels and frailty, but the results indicate the importance of reducing the ratio between the two. In the future, further experimental research is needed to determine whether the anti-inflammatory and antioxidant mechanisms of ω-3 can serve as preventive mechanisms against frailty, as well as to explore the interaction between ω-3 and ω-6 in the frail process.

This work has certain limitations. First, frailty is a dynamic condition that may change over time, and there are complex and variable factors at play. Second, the intake of ω-3 and ω-6 fatty acids is assessed through questionnaire surveys, and their components are defined based on food composition lists, which may not accurately reflect the precision of each component and individuals’ long-term intake levels. However, the large sample size in this study provides a reasonable representation of the average intake levels in the U.S. population. Additionally, as a cross-sectional study with the inability to track respondent information in the NHANES database, this research cannot evaluate the subsequent frailty risk in robust individuals with low ω-3 intake. Long-term tracking of frailty risk in such populations is necessary and warrants further investigation. Lastly, the lack of standardized frailty criteria makes the generalization of the study’s conclusions a more cautious endeavor.

## Conclusion

Our research findings support that daily intake of ω-3 exceeding 2.05 grams and ω-6 intake ranging from greater than 11.423 grams to less than or equal to 19.160 grams is beneficial in reducing frailty risk among middle-aged and elderly individuals. Based on this, we encourage individuals in this demographic in the United States to increase their intake of ω-3 while moderately reducing their intake of ω-6. Additionally, further investigation is warranted into the mechanisms of ω-3’s anti-inflammatory and antioxidant properties in preventing frailty risk, as well as the interaction between ω-3 and ω-6.

## Data availability statement

The data presented in the study are deposited in the Centers for Disease Control and Prevention. The links are as follows: 2005-2006: https://wwwn.cdc.gov/Nchs/Nhanes/2005-2006/DEMO_D.htm and https://wwwn.cdc.gov/Nchs/Nhanes/2005-2006/DR1IFF_D.htm.

## Author contributions

ZY: Conceptualization, Data curation, Methodology, Software, Validation, Writing – original draft. YX: Data curation, Methodology, Validation, Writing – original draft.

KL: Data curation, Methodology, Supervision, Writing – original draft. WZ: Formal analysis, Project administration, Validation, Writing – review & editing. LL: Methodology, Supervision, Validation, Writing – review & editing.
