# Supplementary material for: The relationship between dietary intake of ω-3 and ω-6 fatty acids and frailty risk in middle-aged and elderly individuals: a cross-sectional study from NHANES
Source: Front Nutr. 2024 May 9;11:1377910. doi: 10.3389/fnut.2024.1377910 (PMC11111862; doi:10.3389/fnut.2024.1377910)
Supplement: Supplementary file 1 [file Data_Sheet_1.PDF]

Supplementary material content

1. Table S1. The variables and their respective scores from the 49 Frailty Index used in this study. 2
2. Table S2. Participant characteristics by ω-3 fatty acids intake level. 4
3. Table S3. Participant characteristics by ω-3 supplements intake level. 6
4. Table S4. Participant characteristics by ω-6 fatty acids intake level. 8
5. Table S5. Participant characteristics by ω-6: ω-3 ratio level. 10
6. Table S6. Characteristics of participants by Robust or Frail (Adjusting for lipid-lowering drugs) 12
7. Table S7. Weighted multivariate adjusted logistic regression analysis of frailty risk with different omega intake levels in NHANES from 2005 to 2014 after adjusting for lipid-lowering drugs 14

1. Table S1. The variables and their respective scores from the 49 Frailty Index used in this study.

Item	Score
**Cognition**	Yes = 1, No = 0
1. Experience confusion/memory problems	
**Dependence**	Difficulty = 1, No Difficulty = 0
1. Managing money	
2. Stooping, crouching, kneeling	
3. Lifting or carrying	
4. House chore	
5. Preparing meals	
6. Standing up from armless chair	
7. Getting in and out of bed difficulty	
8. Using fork, knife, drinking from cup	
9. Dressing yourself	
10. Standing for long periods difficulty	
11. Grasp/holding small objects	
12. Attending social event	
13. Push or pull large objects	
14. Walking for a quarter mile difficulty	
15. Walking up 10 steps difficulty	
**Depressive Symptoms**	Nearly every day = 1, More than half the days = 0.66, Several days = 0.33, Not at all = 0
1. Have little interest in doing things	
2. Feeling down, depressed, or hopeless	
3. Trouble sleeping or sleeping too much	
4. Feeling tired or having little energy	
5. Poor appetite or overeating	
6. Feeling bad about yourself	
7. Trouble concentrating on things	
**Comorbidities**	Yes = 1, Suspect = 0.5 No = 0
1. Arthritis	
2. Thyroid problems	
3. Chronic bronchitis	
4. Cancer	
5. Congestive heart failure	
6. Coronary heart disease	
7. Angina	

Item	Score
8. Heart attack	
9. Stroke	
10. Blood pressure	
11. Diabetes	
12. weak/failing kidneys	
13. Urinary Leakage	
**Hospital Utilization and Access to Care**	
1. Self-rated health	Fair, poor = 1, Excellent, Very good, good = 0
2. Health now compared with 1 year ago	Worse = 1, About the same, better = 0
3. Overnight hospital patient in past year	Yes = 1, No = 0
4. Frequency of health care use during past year	None = 0, 1—5 = 0,5, More than 5 = 1
5. Number of prescribed medications	None = 0, 1—5 = 0,5, More than 5 = 1
**Physical Performance and Anthropometry**	
1. Body mass index	
	<18.5, ≥30 = 1
	25—<30 = 0.5
	18.5—25 = 0
2. Handgrip strength	
Male:	For BMI ≤ 24, GS ≤ 29
	For BMI 24.1—28, GS ≤ 30
	For BMI >28, GS ≤ 32 = 1
Female:	For BMI ≤ 23, GS ≤ 17
	For BMI 23.1—26, GS ≤ 17.3
	For BMI 26.1—29, GS ≤ 18
	For BMI>29, GS ≤ 21 = 1
**Laboratory Values**	
1. Glycohemoglobin (%)	0%—5.7% = 0, >5.7% = 1
2. Red blood cell count (million cells/mL)	M: 4.7—6.1 = 0, Other = 1; F: 4.2—5.4 = 0, Other = 1
3. Hemoglobin (g/dL)	M: 13.5—18 = 0, Other = 1; F:12—16 = 0, Other = 1
4. Red cell distribution width (%)	11.6—14.6 = 0, Other = 1
5. Lymphocyte percent (%)	20—40 = 0, Other = 1
6. Segmented neutrophils percent (%)	40—80 = 0, Other = 1

BMI, Body mass index; GS, grip strength; M, Male; F, Female.
This table is based on Hakeem FF's research.

2. Table S2. Participant characteristics by ω-3 fatty acids intake level.

**Characteristic**	**ω-3 Overall**, N = 12315 (100%)[2]	**T1**(≤1.175 g/d) N = 4487 (33%)[1]	**T2**(＞1.175, ≤2.050 g/d) N = 4054 (33%)[1]	**T3**(＞2.050 g/d) N = 3774 (33%)[1]	**P Value**[2]
**Age (years)**	59.9 (10.6)	61.0 (11.0)	60.1 (10.6)	58.6 (10.1)	
**Sex**					**<0.001**
Female	6,305 (53%)	2,669 (63%)	2,076 (54%)	1,560 (43%)	
Male	6,010 (47%)	1,818 (37%)	1,978 (46%)	2,214 (57%)	
**Race**					**0.008**
Non-Hispanic White	6,155 (76%)	2,154 (74%)	2,075 (77%)	1,926 (77%)	
Non-Hispanic Black	2,606 (9.7%)	979 (11%)	807 (8.9%)	820 (9.4%)	
Mexican American	1,678 (5.2%)	689 (5.9%)	535 (4.9%)	454 (4.8%)	
Other Hispanic	1,064 (3.7%)	412 (4.2%)	351 (3.4%)	301 (3.4%)	
Other Race - Including Multi-Racial	812 (5.1%)	253 (4.9%)	286 (5.8%)	273 (5.4%)	
**BMI**					**0.008**
Normal(≥18.5,<25)	3,067 (26%)	1,146 (29%)	992 (26%)	929 (24%)	
Obese(≥30)	4,837 (38%)	1,773 (38%)	1,586 (38%)	1,478 (38%)	
Overweight(≥25,<30)	4,411 (36%)	1,568 (34%)	1,476 (36%)	1,367 (38%)	
**Marital**					**<0.001**
Divorced	7,129 (64%)	2,427 (59%)	2,424 (66%)	2,278 (67%)	
Married	4,764 (34%)	1,884 (38%)	1,506 (32%)	1,374 (31%)	
Never married	422 (2.2%)	176 (2.7%)	124 (2.0%)	122 (1.9%)	
**Serum cotinine**	53 (127)	59 (133)	50 (120)	51 (127)	
**Smoking status**					**<0.001**
Current	2,123 (17%)	812 (20%)	679 (15%)	632 (15%)	
Former	4,037 (32%)	1,389 (30%)	1,335 (32%)	1,313 (34%)	
Never	6,155 (51%)	2,286 (50%)	2,040 (52%)	1,829 (51%)	
**Hypertension**					**0.003**
Yes	7,274 (53%)	2,800 (56%)	2,358 (52%)	2,116 (51%)	
No	5,041 (47%)	1,687 (44%)	1,696 (48%)	1,658 (49%)	
**Hyperlipidemia**					0.14
Yes	10,074 (82%)	3,730 (83%)	3,315 (81%)	3,029 (81%)	
No	2,241 (18%)	757 (17%)	739 (19%)	745 (19%)	
**Diabetes**					0.2
Yes	3,311 (20%)	1,309 (21%)	1,070 (20%)	932 (20%)	
No	9,004 (80%)	3,178 (79%)	2,984 (80%)	2,842 (80%)	
**CVD**					**<0.001**
Yes	10,203 (86%)	3,602 (83%)	3,382 (87%)	3,219 (88%)	

Characteristic	**ω-3 Overall**, N = 12315 (100%)[2]	**T1**(≤1.175 g/d) N = 4487 (33%)[1]	**T2**(＞1.175, ≤2.050 g/d) N = 4054 (33%)[1]	**T3**(＞2.050 g/d) N = 3774 (33%)[1]	**P Value**[2]
No	2,112 (14%)	885 (17%)	672 (13%)	555 (12%)	
**Frail index**					**<0.001**
Robust	8,747 (77%)	2,953 (72%)	2,952 (78%)	2,842 (80%)	
Frail	3,568 (23%)	1,534 (28%)	1,102 (22%)	932 (20%)	
**ω-6**					**<0.001**
T1(≤11.423 g/d)	4,663 (33%)	3,627 (78%)	913 (19%)	123 (2.5%)	
T2(＞11.423, ≤19.160 g/d)	3,956 (33%)	745 (19%)	2,374 (60%)	837 (21%)	
T3(＞19.160 g/d)	3,696 (33%)	115 (3.3%)	767 (20%)	2,814 (76%)	
**ω-6/ω-3**					**<0.001**
Recommended (≤4)	264 (1.9%)	49 (0.9%)	41 (0.8%)	174 (4.1%)	
Mildly high (＞4, ≤10)	7,474 (60%)	2,124 (45%)	2,467 (59%)	2,883 (76%)	
High (＞10, ≤15)	3,534 (30%)	1,619 (37%)	1,265 (33%)	650 (18%)	
Very High (>15)	1,043 (8.5%)	695 (17%)	281 (7.1%)	67 (1.6%)	

1Mean ± SD for continuous; n (%) for categorical

2 chi-squared test with Rao & Scott's second-order correction

3. Table S3. Participant characteristics by ω-3 supplements intake level.

**Characteristic**	**Overall**, N = 656 (100%)[1]	**Robust**, N = 516 (84%)[2]	**Frail**, N = 140 (16%)[2]	**P Value**[3]
**Supplementation (ω-3)**	1.03 (1.70)	1.06 (1.81)	0.85 (0.86)	0.12
**Age (years)**	62.5 (9.7)	61.9 (9.5)	65.5 (10.7)	**0.024**
**Sex**				0.9
Female	345 (53%)	271 (53%)	74 (52%)	
Male	311 (47%)	245 (47%)	66 (48%)	
**Race**				**0.050**
Non-Hispanic White	428 (86%)	346 (88%)	82 (79%)	
Non-Hispanic Black	62 (3.4%)	42 (2.9%)	20 (6.4%)	
Other Race - Including Multi-Racial	60 (4.6%)	48 (3.8%)	12 (7.5%)	
Mexican American	56 (2.8%)	38 (2.5%)	18 (4.5%)	
Other Hispanic	50 (2.8%)	42 (2.8%)	8 (2.6%)	
**BMI**				**<0.001**
Normal(≥18.5,<25)	172 (27%)	146 (29%)	26 (14%)	
Obese(≥30)	231 (34%)	162 (31%)	69 (54%)	
Overweight(≥25,<30)	253 (39%)	208 (40%)	45 (32%)	
**Marital**				0.071
Divorced	448 (72%)	368 (74%)	80 (62%)	
Married	200 (27%)	142 (25%)	58 (37%)	
Never married	8 (0.8%)	6 (0.8%)	2 (0.4%)	
**LBXCOT**	25 (102)	21 (87)	48 (154)	0.14
**Smoking.status**				0.2
Current	54 (7.1%)	36 (6.2%)	18 (12%)	
Former	246 (38%)	196 (38%)	50 (39%)	
Never	356 (55%)	284 (56%)	72 (50%)	
**Hypertension**				**<0.001**
Yes	385 (52%)	272 (47%)	113 (81%)	
No	271 (48%)	244 (53%)	27 (19%)	
**Hyperlipidemia**				0.2
Yes	575 (87%)	452 (87%)	123 (91%)	
No	81 (13%)	64 (13%)	17 (9.1%)	
**Diabetes**				**<0.001**
Yes	154 (19%)	74 (10%)	80 (62%)	
No	502 (81%)	442 (90%)	60 (38%)	
**CVD**				**<0.001**
Yes	542 (86%)	460 (90%)	82 (61%)	

**Characteristic**	**Overall**, N = 656 (100%)[1]	**Robust**, N = 516 (84%)[2]	**Frail**, N = 140 (16%)[2]	**P Value**[3]
No	114 (14%)	56 (9.6%)	58 (39%)	

1Mean ± SD for continuous; n (%) for categorical

2 chi-squared test with Rao & Scott's second-order correction

4. Table S4. Participant characteristics by ω-6 fatty acids intake level.

Characteristic	**ω-6 Overall**, N = 12315 (100%)[1]	**T1** (≤11.423 g/d) N = 4663 (33%)[1]	**T2**(>11.423, ≤19.160 g/d) N = 3956 (33%)[1]	**T3**(>19.160 g/d) N = 3696 (33%)[2]	**P Value**[2]
**Age (years)**	59.9 (10.6)	61.6 (11.2)	59.8 (10.5)	58.2 (9.8)	
**Sex**					**<0.001**
Female	6,305 (53%)	2,821 (65%)	2,034 (54%)	1,450 (41%)	
Male	6,010 (47%)	1,842 (35%)	1,922 (46%)	2,246 (59%)	
**Race**					**<0.001**
Non-Hispanic White	6,155 (76%)	2,153 (72%)	2,074 (78%)	1,928 (78%)	
Non-Hispanic Black	2,606 (9.7%)	970 (10%)	774 (8.6%)	862 (10%)	
Mexican American	1,678 (5.2%)	696 (6.0%)	502 (4.5%)	480 (5.0%)	
Other Hispanic	1,064 (3.7%)	505 (5.0%)	339 (3.4%)	220 (2.6%)	
Other Race - Including Multi-Racial	812 (5.1%)	339 (7.0%)	267 (5.5%)	206 (4.4%)	
**BMI**					**<0.001**
Normal(≥18.5,<25)	3,067 (26%)	1,201 (28%)	1,013 (28%)	853 (23%)	
Obese(≥30)	4,837 (38%)	1,778 (37%)	1,527 (37%)	1,532 (40%)	
Overweight(≥25,<30)	4,411 (36%)	1,684 (35%)	1,416 (36%)	1,311 (37%)	
**Marital**					**<0.001**
Divorced	7,129 (64%)	2,510 (59%)	2,360 (65%)	2,259 (68%)	
Married	4,764 (34%)	1,976 (38%)	1,469 (33%)	1,319 (30%)	
Never married	422 (2.2%)	177 (2.7%)	127 (2.0%)	118 (1.9%)	
**Serum cotinine**	53 (127)	55 (125)	52 (123)	53 (132)	
**Smoking status**					**0.008**
Current	2,123 (17%)	806 (18%)	664 (16%)	653 (15%)	
Former	4,037 (32%)	1,396 (30%)	1,334 (32%)	1,307 (35%)	
Never	6,155 (51%)	2,461 (52%)	1,958 (51%)	1,736 (50%)	
**Hypertension**					**<0.001**
Yes	7,274 (53%)	2,917 (56%)	2,296 (52%)	2,061 (51%)	
No	5,041 (47%)	1,746 (44%)	1,660 (48%)	1,635 (49%)	
**Hyperlipidemia**					0.12
Yes	10,074 (82%)	3,879 (83%)	3,244 (82%)	2,951 (81%)	
No	2,241 (18%)	784 (17%)	712 (18%)	745 (19%)	
**Diabetes**					**0.014**
Yes	3,311 (20%)	1,381 (22%)	1,042 (19%)	888 (19%)	
No	9,004 (80%)	3,282 (78%)	2,914 (81%)	2,808 (81%)	
**CVD**					**<0.001**
Yes	10,203 (86%)	3,751 (83%)	3,305 (87%)	3,147 (88%)	

**Characteristic**	**ω-6 Overall**, N = 12315 (100%)[1]	**T1** (≤11.423 g/d) N = 4663 (33%)[1]	**T2**(>11.423, ≤19.160 g/d) N = 3956 (33%)[1]	**T3**(>19.160 g/d) N = 3696 (33%)[2]	**P Value**[2]
No	2,112 (14%)	912 (17%)	651 (13%)	549 (12%)	
**Frail index**					**<0.001**
Robust	8,747 (77%)	3,106 (72%)	2,882 (78%)	2,759 (80%)	
Frail	3,568 (23%)	1,557 (28%)	1,074 (22%)	937 (20%)	
**ω-3**					**<0.001**
T1(≤1.175 g/d)	4,487 (33%)	3,627 (78%)	745 (19%)	115 (3.3%)	
T2(>1.175, ≤2.050 g/d)	4,054 (33%)	913 (19%)	2,374 (60%)	767 (20%)	
T3(>2.050 g/d)	3,774 (33%)	123 (2.5%)	837 (21%)	2,814 (76%)	
**ω-6/ω-3**					**<0.001**
High (>10, ≤15)	3,534 (30%)	1,192 (27%)	1,223 (31%)	1,119 (31%)	
Mildly high (>4, ≤10)	7,474 (60%)	3,019 (64%)	2,354 (59%)	2,101 (56%)	
Recommended (≤4)	264 (1.9%)	189 (3.8%)	54 (1.4%)	21 (0.6%)	
Very High (>15)	1,043 (8.5%)	263 (5.5%)	325 (8.1%)	455 (12%)	

1Mean ± SD for continuous; n (%) for categorical

2 chi-squared test with Rao & Scott's second-order correction

5. Table S5. Participant characteristics by ω-6: ω-3 ratio level.

Characteristic	**Overall**, N = 12315 (100%)[1]	**High** (> 10, ≤15) N = 3534 (30%)[1]	**Mildly high** (> 4, ≤10), N = 7474 (60%)[1]	**Recommended** (≤4), N = 264 (1.9%)[1]	**Very High** (>15) N = 1043 (8.5%)[1]	**P Value**[2]
**Age (years)**	59.9 (10.6)	59.0 (10.4)	60.4 (10.8)	61.3 (11.0)	58.9 (9.9)	
**Sex**						0.15
Female	6,305 (53%)	1,770 (51%)	3,889 (54%)	145 (57%)	501 (51%)	
Male	6,010 (47%)	1,764 (49%)	3,585 (46%)	119 (43%)	542 (49%)	
**Race**						**<0.001**
Non-Hispanic White	6,155 (76%)	1,795 (77%)	3,718 (76%)	118 (69%)	524 (77%)	
Non-Hispanic Black	2,606 (9.7%)	802 (10%)	1,475 (9.0%)	52 (9.2%)	277 (13%)	
Mexican American	1,678 (5.2%)	558 (5.6%)	950 (5.1%)	19 (3.0%)	151 (5.3%)	
Other Hispanic	1,064 (3.7%)	218 (2.8%)	776 (4.4%)	30 (6.3%)	40 (1.6%)	
Other Race - Including Multi-Racial	812 (5.1%)	161 (4.6%)	555 (5.5%)	45 (12.5%)	51 (3.1%)	
**BMI**						0.071
Normal(≥18.5,<25)	3,067 (26%)	815 (24%)	1,909 (27%)	89 (34%)	254 (27%)	
Obese(≥30)	4,837 (38%)	1,478 (40%)	2,855 (37%)	82 (30%)	422 (38%)	
Overweight(≥25,<30)	4,411 (36%)	1,241 (36%)	2,710 (36%)	93 (37%)	367 (35%)	
**Marital**						0.5
Divorced	7,129 (64%)	2,044 (66%)	4,339 (64%)	147 (63%)	599 (62%)	
Married	4,764 (34%)	1,361 (32%)	2,881 (34%)	108 (35%)	414 (36%)	
Never married	422 (2.2%)	129 (2.1%)	254 (2.3%)	9 (2.1%)	30 (1.9%)	
**Serum cotinine**	53 (127)	56 (128)	50 (123)	55 (127)	64 (145)	
**Smoking status**						**0.027**
Current	2,123 (17%)	664 (18%)	1,200 (16%)	42 (17%)	217 (19%)	
Former	4,037 (32%)	1,139 (31%)	2,443 (33%)	79 (32%)	376 (35%)	
Never	6,155 (51%)	1,731 (51%)	3,831 (52%)	143 (51%)	450 (46%)	
**Hypertension**						0.5
Yes	7,274 (53%)	2,055 (52%)	4,459 (54%)	139 (48%)	621 (53%)	
No	5,041 (47%)	1,479 (48%)	3,015 (46%)	125 (52%)	422 (47%)	
**Hyperlipidemia**						0.8
Yes	10,074 (82%)	2,881 (81%)	6,119 (82%)	216 (81%)	858 (83%)	
No	2,241 (18%)	653 (19%)	1,355 (18%)	48 (19%)	185 (17%)	
**Diabetes**						0.5
Yes	3,311 (20%)	973 (20%)	2,013 (20%)	65 (16%)	260 (19%)	
No	9,004 (80%)	2,561 (80%)	5,461 (80%)	199 (84%)	783 (81%)	
**CVD**						0.3

**Characteristic**	**Overall**, N = 12315 (100%)[1]	**High** (> 10, ≤15) N = 3534 (30%)[1]	**Mildly high** (> 4, ≤10), N = 7474 (60%)[1]	**Recommended** (≤4), N = 264 (1.9%)[1]	**Very High** (>15) N = 1043 (8.5%)[1]	**P Value**[2]
Yes	10,203 (86%)	2,934 (87%)	6,200 (86%)	221 (88%)	848 (85%)	
No	2,112 (14%)	600 (13%)	1,274 (14%)	43 (12%)	195 (15%)	
**Frail index**						0.5
Robust	8,747 (77%)	2,500 (77%)	5,316 (76%)	202 (80%)	729 (77%)	
Frail	3,568 (23%)	1,034 (23%)	2,158 (24%)	62 (20%)	314 (23%)	
**ω-3**						**<0.001**
T1(≤1.175 g/d)	4,487 (33%)	1,619 (42%)	2,124 (25%)	49 (15%)	695 (66%)	
T2(>1.175, ≤2.050 g/d)	4,054 (33%)	1,265 (37%)	2,467 (33%)	41 (14%)	281 (28%)	
T3(>2.050 g/d)	3,774 (33%)	650 (21%)	2,883 (42%)	174 (70%)	67 (6.2%)	
**ω-6**						**<0.001**
T1(≤11.423 g/d)	4,663 (33%)	1,192 (30%)	3,019 (36%)	189 (65%)	263 (22%)	
T2(>11.423, ≤19.160 g/d)	3,956 (33%)	1,223 (35%)	2,354 (33%)	54 (25%)	325 (32%)	
T3(>19.160 g/d)	3,696 (33%)	1,119 (35%)	2,101 (31%)	21 (10%)	455 (46%)	

1Mean ± SD for continuous; n (%) for categorical

2 chi-squared test with Rao & Scott's second-order correction

6. Table S6. Characteristics of participants by Robust or Frail (Adjusting for lipid-lowering drugs)

Characteristic	**Overall**, N = 4437 (100%)[1]	**Robust**, N = 2489 (62%)[2]	**Frail**, N = 1948 (38%)[2]	P Value
**Age (years)**	64.6 (10.2)	64.0 (9.8)	65.6 (10.7)	**<0.001**
**Sex**				**<0.001**
Female	2,078 (48%)	1,068 (44%)	1,010 (55%)	
Male	2,359 (52%)	1,421 (56%)	938 (45%)	
**Race**				**<0.001**
Non-Hispanic White	2,476 (80%)	1,473 (83%)	1,003 (74%)	
Non-Hispanic Black	868 (8.5%)	405 (6.2%)	463 (12%)	
Mexican American	471 (3.7%)	243 (3.0%)	228 (5.1%)	
Other Hispanic	319 (2.8%)	178 (2.7%)	141 (3.0%)	
Other Race - Including Multi-Racial	303 (5.4%)	190 (5.1%)	113 (5.9%)	
**BMI**				**<0.001**
Normal(≥18.5,<25)	800 (17%)	520 (20%)	280 (13%)	
Obese(≥30)	2,076 (47%)	997 (41%)	1,079 (57%)	
Overweight(≥25,<30)	1,561 (36%)	972 (39%)	589 (30%)	
**Marital**				**<0.001**
Divorced	2,683 (67%)	1,660 (72%)	1,023 (58%)	
Married	1,626 (32%)	775 (27%)	851 (39%)	
Never married	128 (1.8%)	54 (1.2%)	74 (2.8%)	
**Serum cotinine**	43 (112)	35 (107)	55 (120)	**<0.001**
**Smoking status**				**<0.001**
Current	616 (13%)	259 (10%)	357 (19%)	
Former	1,809 (40%)	1,007 (39%)	802 (42%)	
Never	2,012 (46%)	1,223 (50%)	789 (39%)	
**Hypertension**				**<0.001**
Yes	3,383 (72%)	1,706 (64%)	1,677 (84%)	
No	1,054 (28%)	783 (36%)	271 (16%)	
**Diabetes**				**<0.001**
Yes	2,069 (39%)	852 (27%)	1,217 (58%)	
No	2,368 (61%)	1,637 (73%)	731 (42%)	
**CVD**				**<0.001**
Yes	2,907 (69%)	1,985 (81%)	922 (49%)	
No	1,530 (31%)	504 (19%)	1,026 (51%)	
**ω-3**	1.90 (1.37)	1.98 (1.39)	1.78 (1.32)	**0.003**
**ω-6**	17 (10)	18 (10)	16 (11)	**<0.001**
**ω-6/ω-3**	10.2 (5.5)	10.1 (5.2)	10.3 (5.9)	0.7

[1]Mean ± SD for continuous; n (%) for categorical; [2]chi-squared test with Rao & Scott's second-order correction. ω-3: omega-3 fatty acids; ω-6: omega-6 fatty acids.

7. Table S7. Weighted multivariate adjusted logistic regression analysis of frailty risk with different omega intake levels in NHANES from 2005 to 2014 after adjusting for lipid-lowering drugs

Regression model	Crude Model<br>OR (95% CI)	Model 1<br>OR (95% CI)	Model 2<br>OR (95% CI)	Model3<br>OR (95% CI)
**ω-3**				
T1	Reference	Reference	Reference	Reference
T2	0.77(0.62, 0.97) **	0.82(0.64, 0.99)*	0.83(0.66, 1.05) **	0.80(0.63, 1.02)
T3	0.70(0.56, 0.88)**	0.76(0.59, 0.96)*	0.80(0.62, 0.98)*	0.78(0.60, 0.96)*
**ω-6**				
T1	Reference	Reference	Reference	Reference
T2	0.70(0.57, 0.87) **	0.75(0.61, 0.92) **	0.77(0.62, 0.96)*	0.77(0.61, 0.97)*
T3	0.68(0.56, 0.84)***	0.77(0.62, 0.97)*	0.77(0.61, 0.97)*	0.81(0.62, 1.04)

*P < 0.05; **P < 0.01; ***P < 0.001

Multiple logistic regression model: Model 1: Adjusted for Age, Sex; Model 2: Adjusted for Age, Sex, Race, Marital, Serum Cotinine, BMI, Smoking status; Model 3: Adjusted for Age, Sex, Race, Marital, BMI, Smoking status, Hypertension, Diabetes. ω-3: omega-3 fatty acids; ω-6: omega-6 fatty acids.
